# Insight into LncRNA- and CircRNA-Mediated CeRNAs: Regulatory Network and Implications in Nasopharyngeal Carcinoma—A Narrative Literature Review

**DOI:** 10.3390/cancers14194564

**Published:** 2022-09-20

**Authors:** Senmiao Zhang, Yanling Li, Shuyu Xin, Li Yang, Mingjuan Jiang, Yujie Xin, Yiwei Wang, Jing Yang, Jianhong Lu

**Affiliations:** 1 Hunan Cancer Hospital/the Affiliated Cancer Hospital of Xiangya School of Medicine, Central South University, Changsha 410013, China; 2 Department of Medical Microbiology, School of Basic Medical Science, Central South University, Changsha 410078, China; 3 Key Laboratory of Cancer Carcinogenesis and Invasion of Chinese Ministry of Education, NHC Key Laboratory of Carcinogenesis, Central South University, Changsha 410078, China; 4 China-Africa Research Center of Infectious Diseases, Central South University, Changsha 410078, China

**Keywords:** long noncoding RNA, circular RNA, microRNA, competing endogenous RNA, regulatory network, nasopharyngeal carcinoma

## Abstract

**Simple Summary:**

Competitive endogenous RNAs (ceRNAs) can regulate gene expression at the posttranscriptional level by competitively binding to microRNAs. In cancer, ceRNA activity is dysregulated. The imbalanced ceRNA-related regulatory network has been theorized to play an important role in cancer progression. In this review, we summarize the biological functions and clinical implications of long noncoding RNAs and circular RNAs as ceRNAs in nasopharyngeal carcinoma (NPC).

**Abstract:**

Nasopharyngeal carcinoma (NPC) is a kind of head-and-neck malignant tumor, and distant metastasis treatment resistance is the leading cause of patient death. In-depth understanding of NPC progression and treatment failure remains to be explored. Long noncoding RNAs (lncRNAs) and circular RNAs (circRNAs) are noncoding RNAs that play key regulatory role in shaping tumor cell activities. Recent studies have revealed that lncRNA and circRNA function as competitive endogenous RNAs (ceRNAs) by regulating the posttranscriptional expression of genes as miRNA baits. The imbalanced ceRNA networks derived from lncRNA/circRNA-miRNA-mRNA interaction are widely found to contribute to NPC development. Herein, we summarize typical examples of lncRNA/circRNA-associated ceRNAs in recent years, which involved the potential molecular mechanisms in the regulation of proliferation, apoptosis, treatment resistance and metastasis of NPC, and discuss their potential clinical significance in the prognosis and treatment of NPC. Interpreting the involvement of ceRNAs networks will provide new insight into the pathogenesis and treatment strategies of NPC. However, ceRNA regulatory mechanism has some limitations currently. Screening the most effective ceRNA targets and the clinical application of ceRNA still has many challenges.

## 1. Introduction

Nasopharyngeal carcinoma (NPC) is a malignancy with high incidence in China that develops on the top and lateral wall of the nasopharynx cavity [1]. The incidence and mortality rate of NPC are 1.7/10^4^ and 1.0/10^4^ in males and 0.7/10^4^ and 0.4/10^4^ in females, respectively [2]. Epstein–Barr virus (EBV) is a causative factor of NPC and participates in the multistage process of NPC [3]. The occurrence of NPC may also be closely related to diet and environment [4,5]. Owing to the complex anatomy of the nasopharynx, the occult nature of the site of NPC, and the atypical early symptoms, approximately 70% of NPC patients already have locally advanced or metastatic disease at the time of diagnosis [6]. Currently, the most effective treatment of NPC is radiotherapy-based comprehensive treatment, but distant metastasis and the weak radiation sensitivity of the tumors may hinder the success of treatment [7,8]. In addition, multidrug resistance (MDR) is the major cause of chemotherapy failure in locally advanced NPC [9]. Cisplatin, paclitaxel, and 5-FU are commonly used chemotherapy drugs [10] that can significantly improve treatment efficiency, but their use in large doses often causes serious cytotoxic reactions and thus induces tumors to develop MDR [11,12,13,14]. Although immune checkpoint inhibitor (ICI) therapy is regarded as a novel standard of care in multiple malignancies like NPC, only a minority of patients benefit from it at present [1]. Hence, finding sensitive and specific biomarkers is a key to improving the cure rate of NPC, achieving an excellent prognosis, and predicting recurrence and metastasis.

Long noncoding RNAs (lncRNAs) are noncoding RNAs (ncRNAs) longer than 200 bp. When initially discovered, lncRNAs were considered to have no biological function [15]. However, recent studies have shown that nuclear lncRNA can interact with DNA [16], RNA [16], protein [17] and other molecules, regulate the chromosome structure [16,17,18] and participate in chromatin reconstruction. Completely processed lncRNAs are transported to the cytoplasm or other organelles, and cytoplasmic lncRNAs act as posttranscriptional regulators to directly target mRNA transcripts to regulate their stability or inhibit mRNA through a competitive endogenous RNA (ceRNA) mode [19,20]. Extensive studies have confirmed that lncRNAs, as ceRNAs, are widely involved in the occurrence and development of NPC. This review summarizes this aspect next.

As a newly discovered type of noncoding RNA molecule, circular RNA (circRNA) forms a closed loop by backsplicing to protect it from ribonuclease R and it is stably expressed [21]. Based on the origin of its sequence, circRNAs can be divided into three categories: exonic circRNAs, intronic circRNAs, and exon–intron circRNAs [22]. In the past, circRNA was thought to be a useless by-product of incorrect splicing [23]. Recent studies have found that circRNA is an important participant in the development of cancer. Some circRNAs contain open reading frames (ORFs) and have protein-coding functions [24]. CircRNAs usually contain several microRNA (miRNA) binding sites, and some contain one or multiple RNA binding sites, which can be used as sponges for miRNA. As lncRNAs, circRNAs are also able to function as ceRNA via miRNA response elements in them. Increasing evidence has shown that circRNAs participate in proliferation, metastasis, and invasion and even affect chemotherapy resistance and radiosensitivity of NPC by acting as ceRNAs [25,26,27].

**Figure 1:**
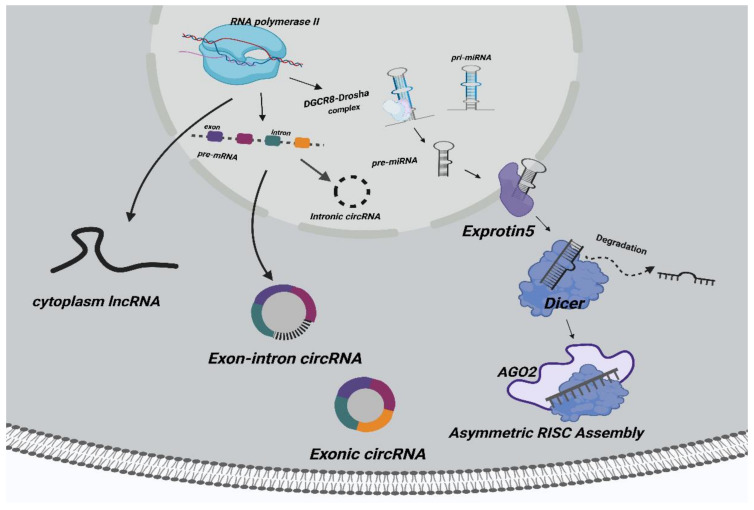
The synthetic pathways of noncoding RNAs. Cytoplasmic lncRNAs are transcribed from its corresponding parent gene. CircRNAs are reverse-spliced from precursor mRNAs and form three types, namely, exon circRNAs, intron circRNAs, and exon–intron circRNAs. MiRNAs are transcribed by RNA polymerase II to generate pre-miRNA and then cut into pre-miRNA through the DGCR8–Drosha enzyme complex. Exprotin 5 transports pre-miRNA out of the nucleus. Dicer and other enzymes cut pre-miRNA into unstable double-stranded RNA outside of the nucleus. One single-stranded miRNA is degraded, and the other is integrated into RISC (ribonuclein complex containing members of AGO histones).

In eukaryotes, some small RNA molecules are often used to guide gene silencing, such as small interfering RNA (siRNA) and miRNA, a mechanism known as RNA interference (RNAi) [28]. MiRNA is also a kind of noncoding RNA with a length of about 20 bp, which is a highly conserved gene family [29]. Studies have shown that the RNA-induced silencing complex (RISC) is the key to miRNA function, and it is comprised of siRNA, the Dicer enzyme, Argonaut protein (AGO), and other biological macromolecules [30]. Based on its complementary pairing form with mRNA, there are two functions of miRNA: complete complementary pairing, where RISC degrades the mRNA, and incomplete complementary pairing, which inhibits mRNA translation [31]. Researchers have confirmed that miRNAs play biological roles in tumors by negatively regulating downstream genes [32,33,34]. MiRNAs are considered to be promising targets for cancer therapy. Given that miRNAs are key points in the ceRNA networks, lncRNAs and circRNAs serving as ceRNAs may therefore serve as potential therapeutic targets. The synthesis process of noncoding RNAs (lncRNAs, circRNAs, miRNAs) is shown in Figure 1.

The ceRNA hypothesis was proposed by the Pier Paolo Pandolfi research group of Harvard Medical College in 2011 [20]. There are one or more mRNA binding sites on miRNAs that degrade target genes or inhibit translation through RISC [35]. However, some lncRNAs/circRNAs form complementary pairing with miRNAs as ceRNAs to competitively bind miRNAs, thereby preventing miRNAs from inhibiting their target genes. The ceRNA hypothesis has attracted wide attention since it was first proposed, and it may explain the biological role of some lncRNAs and circRNAs. Significantly, many studies have confirmed that the ceRNA mode exists in a variety of cancers [36,37,38]. In recent years, emerging studies have showed that lncRNA- and circRNA-mediated ceRNA networks play an important role in NPC progression.

## 2. Methods

We searched several databases to identify relevant studies: Google Scholar, PubMed, and Medline. Search terms such as “ceRNA,” “NPC,” “non-coding RNA,” “lncRNA,” and “circRNA” were used in various combinations to obtain relevant information. We collected research papers on the ceRNA mechanism involved in NPC progression and integrated information to determine the content of the narrative review. Date restrictions (published ≥2017) were only used to obtain and summarize evidence of popular literature.

## 3. Biological Functions of LncRNA/CircRNA-Mediated CeRNA Networks in NPC

Accumulating evidence from studies has shown that lncRNA and circRNA participate in the occurrence and development of various cancers by adsorbing miRNAs to inhibit the expression of target genes. As a novel mode of gene regulation, ceRNA reveals the potential function of ncRNAs. In this section, we review lncRNA/circRNA-miRNA-target gene networks regulating biological functions of NPC.

### 3.1. Regulation of NPC Cell Proliferation

Tumor cells have self-sufficient proliferative capacity, even without external stimulation. The ceRNA mechanism is one of the ways to maintain proliferation signal transduction in NPC cells. Almost all lncRNAs/circRNAs function as ceRNAs, which can promote the proliferation of NPC tumor cells. We focus on ceRNA networks that maintain the proliferation signal transduction ability of NPC tumor cells. Their specific molecular mechanisms are shown in Table 1 and Table 2.

For instance, tumor cells increase de novo fatty acid synthesis to promote proliferation. Oncogenic LNC02570 is upregulated in advanced stage NPC patients. High expression of LINC02570 significantly promotes NPC proliferation. As a ceRNA, LINC02570 upregulates the expression level of the key gene SREBP1 in the lipid biosynthesis pathway by adsorbing miR-4649-3p [47]. SREBP1 simultaneously activates FASN (downstream gene) expression. In previous studies, EBV-LMP1 mediated the activation of the SREBP1-FASN pathway, an important mechanism for promoting NPC proliferation [59]. LINC02570, as a ceRNA, plays the same function as LMP1. Although the correlation between LMP1 and LINC02570 has not been reported, LINC02570 shows a new function of ceRNA in the activation of lipogenesis, an effect that accelerates NPC proliferation and tumor development. In tumor cells, lipogenesis is highly active and promotes the rapid proliferation of tumors. Hence, targeting lncRNA or key genes for lipogenesis in ceRNA networks may effectively inhibit the proliferation of NPC cells.

The atypical cell cycle process caused by abnormal activation of the PI3K/Akt signaling pathway is considered a basic feature of many malignant tumors, including NPC. Excessive activation of this signaling pathway leads to abnormal cell proliferation. CeRNA networks at least partially promote NPC proliferation by inhibiting PTEN expression. PTEN is a tumor suppressor of NPC and a key negative regulatory gene of PI3K/Akt signaling pathway [60,61].

Low expression of PTEN predicts poor prognosis and shorter progression-free survival in NPC patients [61]. Overexpression of circITCH significantly inhibits NPC cell proliferation [58]. In vitro, circITCH blocks miR-214-mediated inhibition of PTEN, suggesting that circITCH, as a ceRNA, may inhibit the PI3K/Akt signaling pathway [58] and NPC proliferation. PTEN can also be upregulated by lncRNA MEG3/miR-21 and promote autophagy and apoptosis in NPC cells [53]. Notably, circITCH and lncRNA MEG3, as ceRNAs, jointly regulate PTEN cells and promote proliferation in HK-1 cells.

Therefore, lncRNA/circRNA-associated ceRNA cross talk may be a potential therapeutic target for NPC. Understanding the specific mechanism of the ceRNA network involved in the regulation of proliferation-related genes and signaling pathways in NPC is of great significance for the development and treatment of NPC.

### 3.2. Regulation of NPC Cell Apoptosis

Apoptosis is a programmed death, which plays a vital role in the development of organisms and defense against intracellular infection factors. Contrary to the effect of cell proliferation, most ceRNA-mediated abnormal expression of apoptosis-related genes inhibits apoptosis of NPC tumor cells and promotes their survival. B cell lymphoma 2 (Bcl-2) is an antiapoptotic protein. It inhibits apoptosis by regulating mitochondrial outer membrane and preventing the release of proapoptotic molecules into the cytoplasm. Bcl-2 is the main target of apoptosis molecular mechanism research, and is also the target gene of lymphoma and other cancer treatment. Some researchers have confirmed that Bcl-2 plays an important role in the antiapoptotic activity of EBV latent proteins (such as BHRF1, BARF1, and EBNA-3C) in the development of NPC. Xue et al. confirmed that the abnormal expression of Bcl-2 induced by lncRNA NEAT1/miR-129 was the main reason for suberoylanilide hydroxamic acid (SAHA)-induced apoptosis [54]. Inhibition of lncRNA NEAT1 and restoration of miR-129 levels are potential therapeutic targets for NPC and strategies for overcoming SAHA resistance. In lncRNA/circRNA mediated ceRNA networks, other apoptosis-related genes such as caspase, Fas and p53 have not been reported.

### 3.3. Modulating NPC Chemosensitivity

Improving the radiosensitivity of NPC is a challenge clinically. CircRNA_000543 increased in radiation-resistant NPC tissue, and the overall survival of patients was poor [26]. CircRNA_000543/miR-9/PDGFRB is a ceRNA involved in NPC radiation resistance, and any element imbalance will affect radiation resistance [26]. Notably, imatinib, a PDFGRB inhibitor used to treat chronic myeloid leukemia (CML), can effectively increase the radiosensitivity of NPC [26]. Elements in circRNA_000543-mediated ceRNA model are potential targets for NPC therapy.

The Gene Expression Omnibus (GEO) database was used to identify lncRNAs with abnormally high PTPRG-AS1 expression. LncRNA PTPRG-AS1 promotes radiosensitivity of NPC cells as a ceRNA [51]. LncRNA PTPRG-AS1 adsorbs miR-194-3p and miR-124-3p negatively regulate PRC1 and LHX2, respectively [50,51]. LHX2 activates the Notch pathway and reduces radiosensitivity of NPC cells. Although the specific mechanism of LncRNA PTPRG-AS1 as a ceRNA involved in radiation resistance of NPC remains unclear, these two studies provided potential biomarkers for evaluating the results of radiation resistance in NPC treatment. Moreover, lncRNA ZFAS1/miR-7-5p regulates ENO2 and participates in NPC radioresistance [39]. Studies have confirmed that ENO2 shapes the hypoxic environment of tumors by regulating HIF-1 signaling, which may be the reason for ZFAS1 as a ceRNA to promote NPC radiation resistance [39]. Furthermore, the lncRNA XIST/miR-381-3p/NEK5 network promotes glycolysis and NPC progression under hypoxic conditions. Oxygen-enriched tumor cells are more sensitive to radiotherapy than hypoxic tumor cells. The above studies show that inhibiting lncRNA-mediated ceRNA networks can effectively regulate the glycolysis and HIF-1 signal of NPC tumor cells to resist low radiosensitivity under hypoxia.

Recent studies have confirmed that lncRNA/circRNA-mediated ceRNA networks play an important role in regulating drug resistance of NPC cells (Table 3). The treatment of recurrent NPC is mainly multidrug chemotherapy containing cisplatin. Cisplatin drugs promote tumor cell death by blocking DNA polymerase and inhibiting gene replication. Compared with parental NPC cells, lncRNA HOXA11-AS is upregulated and miR-454-3p downregulated in cisplatin-resistant NPC cells [62]. Liu et al. confirmed that HOXA11-AS as a ceRNA enhanced cisplatin resistance of NPC cells by adsorbing miR-454-3p [62]. At the same time, the downstream c-Met/Akt/mTOR pathway was activated by HOXA11-AS/miR-454-3p, thereby promoting the cisplatin resistance of NPC cells [62]. Combined use of Si-HOXA11-AS and cisplatin may improve the treatment of drug-resistant NPC. As a ceRNA, lncRNA MAGI2-AS3 confers cisplatin resistance to NPC cells by regulating miR-218-5p/GDPD5 [63]. Furthermore, circNRIP1 was identified as the oncogene of multiple tumors [64,65]. It was highly expressed in sera of chemotherapy-resistant NPC patients [27,64]. The knockdown of CircNRIP1 significantly enhanced the sensitivity of NPC cells to 5-Fu and cisplatin by competitive binding with miR-515-5p [27,64]. Targeted inhibition of circNRIP1-mediated ceRNA model is a potential therapeutic strategy for drug-resistant NPC patients [27,64].

Paclitaxel is also one of the commonly used chemotherapy drugs for recurrent NPC. It inhibits tumor cell division by promoting tubulin polymerization and inhibiting its depolymerization. CPEB2 is an RNA-binding protein, and its role in tumors is contradictory. The identical CPEB2A of CPEB2 is a tumor suppressor and inhibits the translation of target mRNA, while the identical CPEB2 can competitively bind to the target RNA of CPEB2A, thus allowing subsequent translation. CPEB2B is a tumor promoter [68,69]. LncRNA CCAT1, as a ceRNA, regulates CPEB2 at posttranscriptional level. In NPC, it seems to be a tumor-promoting factor. The specific mechanism of CPEB2 involved in promoting paclitaxel resistance in NPC cells is worthy of further study, which provides guidance for the potential regulatory function of ceRNA. Paclitaxel is sensitive to G2/M cells and significantly promotes the radiosensitivity of tumor cells. Targeted inhibition of a CCAT1-mediated ceRNA model may promote the therapeutic effect of radiotherapy and chemotherapy in paclitaxel resistant NPC patients.

### 3.4. Modulating NPC Metastasis

After chemoradiotherapy, 20–30% of NPC patients still face metastasis [70]. Their median overall survival (OS) time was shortened to 10–20 months [71]. Therefore, effective molecular targets are urgently needed for the treatment of metastatic NPC. Some lncRNAs/circRNAs act as ceRNAs and are involved in the regulation of NPC cell invasion, migration and metastasis, which were also verified in vivo experiments (Table 4). These lncRNAs/circRNAs may provide promising cancer biomarkers for early diagnosis and prognosis in patients with metastatic NPC.

Epithelial–mesenchymal transition (EMT) is a process in which epithelial cells lose their characteristics and acquire mesenchymal characteristics. In cancer, EMT is considered to be the key mechanism in metastasis. EMT is often regarded as a binary process of epithelial cells and mesenchymal cells [76].

TGF-β is considered to be the most important signaling pathway of EMT induced by tumor cells. TGF-β-mediated EMT can occur in two ways. One is TGF-β signaling activates Smad2 and Smad3, and then forms a complex with Smad4 to transfer into the nucleus and mediates the inhibition and activation of target genes with EMT-related transcription factors (TF). Snail, Slug, and ZEB1 were identified as the main transcription activators of EMT. The loss of epithelial gene E-cadherin is the marker of EMT, and the upregulation of mesenchymal gene N-cadherin is also the key phenomenon of EMT [77]. In addition, TGF-β can induce EMT together with Notch, Wnt, and other signaling pathways [78,79].

Recent studies have shown that lncRNA/circRNA-mediated ceRNA networks induce tumor EMT formation by activating TGF-β signals, e.g., lncRNA AATBC regulates PNN by adsorbing miR-1237-3p. The interaction between PNN and ZEB1 can also promote EMT of NPC [72]. Circ_0046263 promotes lymph-node metastasis of NPC cells in vivo [74]. The potential regulatory mechanism is to relieve the inhibition of miR-133a-5p on IGFBP3 expression and promote IGFBP3-mediated TGF-β activation [74].

In addition, the reconstruction of the cytoskeleton can also trigger EMT formation. CircRNA participates in the assembly of the dynamic cytoskeleton through a ceRNA mechanism, thereby promoting metastasis in NPC. CircSETD3 competitively adsorbs miR-615-5p and miR-1538, and ultimately upregulates MAPRE1 [25].The upregulation of MAPRE1 prevents α-tubulin acetylation, thereby affecting NPC cell motility, migration and EMT [25]. CircSETD3 was once considered a tumor suppressor; however, ceRNA mechanism conferred it an oncogene function [80]. It provides a new potential biomarker for metastatic NPC.

Virus ncRNA can also be used as miRNA decoys to participate in ceRNA network regulation. This regulation pattern has been defined as “competitive viruses and host RNA” (cvhRNAs) [81]. This novel interaction was first described in the hepatitis B virus (HBV) ncRNA and later in human papilloma (HPV) [82,83]. EBV-encoded circRNAs can also serve as ceRNAs by adsorbing host miRNAs, thereby hindering the targeted inhibition of miRNAs on target genes and accelerating the progression of NPC. The EBV-encoded gene RPMS1 exons 2–4 form circRPMS1 through backsplicing from exon 4 to exon 2 (circRPMS1_E4_E2). In EBV-positive NPC, circRPMS1 is activated and sponges multiple miRNAs (miR-203, miR-31, miR-451) to promote the EMT process of NPC and play a role in promoting cancer [57]. Notably, there are very few studies on the involvement of EBV-encoded lncRNAs and circRNAs in the cvhRNA hypothesis. In-depth study of the specific molecular mechanisms of EBV-encoded lncRNAs/circRNAs-mediated ceRNA networks contributes to virus-targeted therapy of NPC.

**Figure 2:**
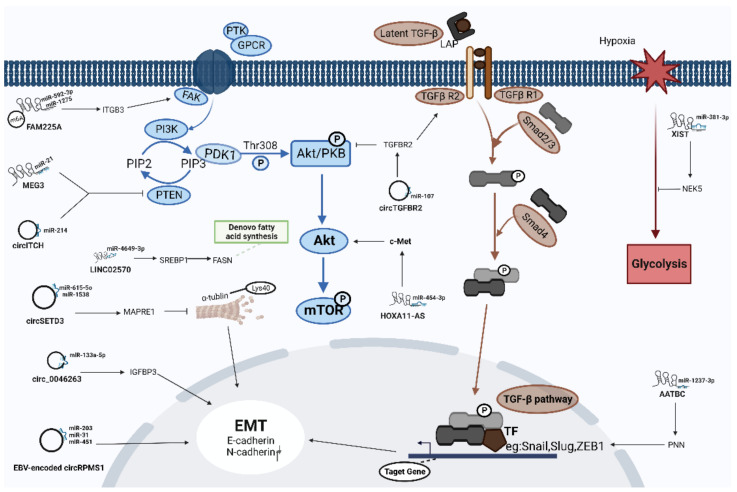
The specific regulatory mechanism of some representative lncRNA/circRNA-mediated ceRNA networks in NPC progression.

Understanding the ceRNA networks mediated by lncRNAs and circRNAs can provide valuable insights into the molecular mechanisms underlying the pathogenesis of a variety of human malignancies, including NPC (Figure 2).

## 4. Implications of LncRNA/CircRNA-Associated ceRNAs as Diagnostic Markers or Therapeutic Targets in NPC

The TNM (tumor–lymph node–metastasis) staging system is a general guideline for cancer treatment. In clinical practice, it cannot accurately identify the risk of recurrence or distant metastasis in NPC patients. Stage II patients are generally considered at low risk. Final recurrence occurs in 15–20% of patients without adjuvant chemotherapy. Conversely, more than half of patients in stage III and IV were cured only by chemoradiation without adjuvant chemotherapy [84]. Thus, novel prognostic biomarkers are needed. The ceRNA spectrum in cancer cells is different from that in normal cells. Some functional ceRNAs are deactivated in tumor cells and may serve as diagnostic or therapeutic markers in the future. For example, lncRNA FOXD3-AS1 as a ceRNA promotes FOXD3 gene expression. The upregulation of these two genes was associated with TNM stage and histological type of NPC [52]. LncRNA FAM225A was identified as an oncogenic gene in NPC. FAM225A, as a ceRNA, effectively activates the FAK/PI3K/Akt signaling pathway by upregulating ITGB3, thereby promoting NPC proliferation and metastasis [45]. The combination of TNM staging and FAM225A expression is expected to become an effective prognostic indicator.

CircCRIM1, as a ceRNA, plays an important prognostic role in NPC metastasis and chemotherapy resistance [38]. Hong et al. combined circCRIM1 expression with N staging to construct a prognostic prediction model [38]. Patients with low CircCRIM1 expression or early-stage N were sensitive to systemic induction chemotherapy of docetaxel, while patients with high CircCRIM1 expression or late-stage N did not benefit from systemic induction chemotherapy of docetaxel [38]. These findings are used clinically to avoid unnecessary drug toxicity.

Knockdown of oncogenic lncRNAs/circRNAs may be an effective strategy to interfere with the progression of NPC, but how to deliver therapeutic nucleic acids accurately into cells based on the ceRNA model is a challenge. Based on the CRISPR/Cas system, the precision of lipid nanoparticles and polymer hydrogel nanoparticles to treat NPC may be a potential solution [85,86].

In a word, miRNAs are considered promising targets for cancer therapy. Given that miRNAs are key points in ceRNA networks, lncRNAs and circRNAs serving as ceRNAs may therefore serve as potential therapeutic targets.

## 5. Conclusions

Since lncRNAs and circRNAs combine noncoding RNAs with protein-coding RNAs through complex ceRNA networks, they play indispensable regulatory roles in cancers like NPC. Exploring the specific molecular mechanism of lncRNAs/circRNAs as ceRNAs could provide targeted molecular therapy and clinical prevention strategies in NPC. Emerging studies have indicated that a variety of dysregulated lncRNA/circRNA-mediated ceRNAs may form networks to regulate multitudinous biological functions in NPC, including tumor cell proliferation, apoptosis, invasion, migration, metastasis, and treatment resistance.

In fact, any transcript containing miRNA seed matches can function as a ceRNA. However, in the complex ceRNA networks, how to select an effective ceRNA as a target and how to specifically manipulate the target ceRNA without affecting other ceRNAs for the treatment of NPC? This is a challenging task for clinical applications of ceRNA. Moreover, the occurrence of NPC is closely related to EBV infection. Although some individual EBV-encoded circRNAs have been confirmed as ceRNAs to accelerate NPC progression, research on EBV-encoded lncRNAs/circRNAs as ceRNAs is still rare in NPC. Targeting EBV-encoded genes is also a specific therapeutic strategy for NPC. All in all, further understanding of the regulatory networks of lncRNA/circRNA-mediated ceRNAs originating from both tumor cells and EBV will provide more novel insight into the pathogenesis and therapy of NPC.

## Figures and Tables

**Table 1:** CeRNA networks of lncRNA-miRNA-mRNA involved in NPC proliferation and apoptosis.

LncRNA	miRNA	mRNA	Function	Reference
ZFAS1	miR-7-5p	ENO2	Proliferation, apoptosis, radiation resistance	[39]
SNHG5	miR-1179	HMGB3	Proliferation, migration and invasion	[40]
SNHG7	miR-514-5p	ELAVL1	Proliferation, migration	[41]
DRAIC	miR-122	SATB1	Proliferation, migration and invasion	[42]
SOX2-OT	miR-146b-5p	HNRNPA2B	Proliferation, apoptosis, migration, invasion and metastasis	[43]
XIST	miR-148a-3p	ADAM17	Proliferation, apoptosis, migration, invasion, EMT and metastasis	[44]
FAM225A	miR-590-3p miR-1275	ITGB3	Proliferation, migration, invasion, metastasis and FAK/PI3K/AKT pathway	[45]
CYTOR	miR-613	ANXA2	Proliferation, migration, invasion and metastasis	[46]
LINC02570	miR-4649-3p	SREBP1	Proliferation, invasion, and migration	[47]
HOXC13-AS	miR-383-3p	HMGA2	Proliferation, invasion, and migration	[48]
SMAD5-AS1	miR-106a-5p	SMAD5	Proliferation, invasion, migration and EMT	[49]
PTPRG-AS1	miR-194-3p	PRC1	Proliferation, apoptosis, invasion, migration, metastasis and radiosensitivity	[50]
PTPRC-AS1	miR-124-3p	LHX2	Proliferation, apoptosis and radiosensitivity	[51]
FOXD3-AS1	miR-185-3p	FOXD3	Proliferation, invasion, migration and cell stemness	[52]
MEG3	miR-21	PTEN	Apoptosis and autophagy	[53]
NEAT1	miR-129	Bcl-2	Apoptosis in SAHA tolerance NPC cell lines	[54]

**Table 2:** CeRNA networks of circRNA-miRNA-mRNA involved in NPC proliferation and apoptosis.

CircRNA	miRNA	mRNA	Function	Reference
CircCTDP1	miR-320b	HOXA10	Proliferation, invasion, migration, apoptosis and TGFβ2 pathway	[55]
CircRNA_000543	miR-9	PDGFRB	Proliferation, apoptosis and radiosensitivity	[26]
CircHIPK3	miR-4288	ELF3	Proliferation, invasion, and migration	[56]
CircTGFBR2	miR-107	TGFBR2	Proliferation, invasion, migration, EMT, TGF-β and PI3K/Akt pathway	[57]
CircITCH	miR-214	PTEN	Proliferation, migration and invasion	[58]

**Table 3:** CeRNA networks of lncRNA/circRNA-miRNA-mRNA involved in NPC chemosensitivity.

LncRNA/CircRNA	miRNA	mRNA	Function	Reference
CCAT1	miR-181a	CPEB2	Paclitaxel resistance	[66]
MAGI2-AS3	miR-218-5p	GDPD5 SEC61A1	Cisplatin resistance and EMT Proliferation and migration	[63]
CircNRIP1	miR-515-5p	IL-25	5-Fu and cisplatin resistance	[27]
CircCRIM1	miR-422a	FOXQ1	Docetaxel chemosensitivity, invasion, migration, metastasis and EMT	[38]
XIST	miR-381-3p	NEK5	Glycolysis, migration, invasion and metastasis under hypoxic conditions	[67]
HOXA11-AS	miR-454-3p	c-Met	Cisplatin resistance, C-Met/AKT/mTOR pathway	[62]

**Table 4:** CeRNA networks of lncRNA/circRNA-miRNA-mRNA involved in NPC metastasis.

LncRNA/CircRNA	miRNA	mRNA	Function	Reference
AATBC	miR-1237-3p	PNN	Migration, invasion, EMT and metastasis	[72]
AFAP1-AS1	miR-423-5p	FOSL2 RAB11B LASP1	Invasion, migration, metastasis and Rho/Rac pathway, invasion	[73]
CircSETD3	miR-615-5p miR-1538	MAPRE1	Invasion, migration and metastasis	[25]
Circ_0046263	miR-133a-5p	IGFBP3	Proliferation, invasion, EMT and metastasis	[74]
EBV-encoded CircRPMS1	miR-203 miR-31 miR-451		Proliferation, invasion, EMT and metastasis	[75]
